# Green Synthesized Silver Nanoparticles Using Tridax Procumbens for Topical Application: Excision Wound Model and Histopathological Studies

**DOI:** 10.3390/pharmaceutics13111754

**Published:** 2021-10-21

**Authors:** Farhat Fatima, Mohammed F. Aldawsari, Mohammed Muqtader Ahmed, Md. Khalid Anwer, Maimuna Naz, Mohammad Javed Ansari, Abubaker M. Hamad, Ameeduzzafar Zafar, Mohammed Jafar

**Affiliations:** 1 Department of Pharmaceutics, College of Pharmacy, Prince Sattam Bin Abdulaziz University, P.O. Box 173, Al-Kharj 11942, Saudi Arabia; moh.aldawsari@psau.edu.sa (M.F.A.); m.anwer@psau.edu.sa (M.K.A.); mj.ansari@psau.edu.sa (M.J.A.); 2 Department of Pharmacology, College of Pharmacy, Prince Sattam Bin Abdulaziz University, P.O. Box 173, Al-Kharj 11942, Saudi Arabia; m.naz@psau.edu.sa; 3 Basic Sciences Department, Preparatory Year Deanship, Prince Sattam Bin Abdulaziz University, P.O. Box 20337, Al-Kharj 11942, Saudi Arabia; a.hamad@psau.edu.sa; 4 Department of Pharmaceutics, College of Pharmacy, Jouf University, Sakaka 72341, Saudi Arabia; azafar@ju.edu.sa; 5 Department of Pharmaceutics, College of Clinical Pharmacy, Imam Abdulrahman Bin Faisal University, P.O. Box 1982, Dammam 34212, Saudi Arabia; mjomar@iau.edu.sa

**Keywords:** green synthesis, histopathology, leaf extract, silver nanoparticles, tridax procumbens, wound healing

## Abstract

The objective of this study was to synthesize silver nanoparticles from the leaves of Tridax procumbens and develop its topical gels using chitosan to investigate the wound healing efficacy concomitant with the histopathological study. Green synthesized silver nanoparticles (AgNPs) were prepared by reacting silver nitrate (0.3 M) with leaf extract and characterized by particle analysis, FTIR, XRD, SEM, BET, and TGA. The results revealed formed AgNPs were nano-sized (138 ± 2.1 nm), monodispersed (PDI: 0.460 ± 0.3), inter-particle repulsion (zeta: −20.4 ± 5.20 mV), stabilized, crystalline and, spherical with size ranging from 80–100 nm as per SEM micro photos. The BET analysis of AgNPs presents the surface area (12.861 m^2^/g), pore volume (0.037 cc/g), and pore radius (24.50 nm).TGA results show a loss of 13.39% up to 300 °C. The topical formulation was developed by loading AgNPs in chitosan-based gels, evaluated by pH, thermal cycling, centrifugal, and spreadability tests. AgNPs chitosan gels results showed skin compatibility, higher stability, and spreading ability. The maximum antibacterial zone of inhibition was found to be 25 ± 0.98 mm for bacillus subtitles and 30 ± 1.99 mm for Klebsiella pneumoniae, respectively. Nanosilver-containing gel also showed excellent compatibility with erythrocytes. Excision wound model was used to assess the wound healing property of the developed AgNP gels, the results of which indicated a significantly progressive healing process in test-group of animals treated with chitosan-based gels containing AgNPs. A histopathological study further confirmed the almost normal skin structure of treated animal tissue compared to standard and negative control. Thus, green synthesized AgNPs loaded chitosan-based topical gel can potentially be used for wound healing application.

## 1. Introduction

Nanotechnology is a science dealing with the materials on a supramolecular scale of 1 and 100 nanometers applied in various applications ranging from industrial to environmental conservation [1]. Nanomedicine, the implication of nanotechnology to medical sciences, has a revolutionary impact on therapeutic and diagnostics management. Nanomaterials have the potential to improve the efficiency of site-specificity and effectiveness against the mutant and pathogens. The most immediate challenge in nanomedical research may be the design and development of multifunctional nanoparticles. Pharmaceutical applications of nanomaterials are extensively reported for site-specific, temporal, and controlled drug release. Nanocarriers discovered thus far include nanoemulsion, nanoparticles, solid lipid nanoparticles, gold, and silver nanoparticles. Silver nanoparticles (AgNPs) possess excellent antibacterial properties, damage the bacterial cell membrane structure, eventually causing apoptosis. AgNPs are extensively used for antimicrobial and antitumor activity. Besides these, their application accelerates the wound healing process. [2]. In the recent past, silver nanoparticles (AgNPs) have been popularly employed in everyday life. There are about 400 commercial products presently using AgNPs in everything from bandage textile coating to wound healing and many more [3]. AgNPs promote wound contraction by differentiation of fibroblasts into myofibroblasts. Its efficacy of epidermal re-epithelialisation fastens the rate of wound closure. Reduction in the wound inflammation contributed by the antibacterial activity of AgNPs. These antibacterial effects of AgNPs could be due to the action of silver ion (Ag+); disruption and perforation of the bacterial cell wall, denaturation of membrane and ribosomes, protein synthesis inhibition, interruption, and interference of adenosine triphosphate (ATP) production and deoxyribonucleic acid (DNA) replication, respectively. AgNPs reported diameters are usually ≤100 nm, encompass about 20 to 15,000 silver (Ag) atoms due to their enormous surface-to-volume ratio, exhibit significant antimicrobial effects even at a low concentration [4,5,6].

A wound is a breakdown of the protective function of the skin and or loss of underlying connective tissue alongside the loss of epithelium continuity caused due to injury of the skin by surgery, ulceration, cancer, wear, and tear. Wound healing is a complex process subdivided into at least three continuous and overlapping steps: inflammation, proliferation leading to tissue restoration, and remodeling [7,8]. Multifaceted application of AgNPs demands huge production, which can be accomplished by diversified synthesis technologies. Physical technologies to produce AgNPs include; evaporation-condensation, electrical irradiation, and gamma irradiation methods. The chemical approach for the synthesis of AgNPs is by reduction mechanism using organic and inorganic reducing agents. Biosynthesized AgNPs employing microorganisms and green synthesized AgNPs applying plant extracts. Green synthesis is the holistic approach to produce the product by avoiding the formation of harmful byproducts. It is a reproducible, reliable, sustainable synthesis technique. Green synthesis is comprehensively employed in one of the evolving nanotechnology techniques for the cost-effective, environmentally friendly, scalable production of AgNPs without the usage of high pressure, temperature, and toxic chemicals [9]. In the recent past, hundreds of plants reportedly used as effective reducing agents to synthesize AgNPs.

Interestingly, green synthesized/prepared plant-based AgNPs represent high yield and stability. Moreover, green synthesis techniques seem to be simple, rapid, non-toxic approaches that can produce well-defined, organized, and definite morphology of nanoparticles [10]. Therapeutics agents obtained from plant sources are being exploited since ancient times as alternative medicine. Tridax procumbens L. (T.P), a native to tropical America, taxonomically classified in the Asteraceae family it is a perennial herb found as a weed throughout the tropical and subtropical regions [11]. A hispid, procumbent herb commonly called ‘Common button’ or ‘Coat button’ is used by traditional health practitioners and tribal communities as a remedy for various ailments and skin diseases. Phytoconstituent of Tridax procumbens comprise β-sitosterol, carotenoids, luteolin, and linolenic acid, among the other active constituents. Beta-sitosterol, a plant steroid, promotes epithelialization and wound healing [12]. Tridax procumbens has been reported to have antimicrobial, antibiotic, antiviral, antifungal, antimalarial, anti-candidal, anti-cancerous, analgesic, anti-inflammatory, antipyretic, antidiabetic, antioxidant, insecticidal, immunomodulatory, and effective against boils, blisters, and cuts [13]. Leaf extract was proved to accelerate the blood clotting sequence and thus helps in hemostasis. It is traditionally known for its dose-dependent wound healing property. Many scientific studies reported Tridax procumbens-based AgNPs elicited significant antibacterial, antioxidant, anticancer, and other allied pharmacological activities [14]. To a greater extent AgNPs, considered to be a double-edged sword despite their tremendous implication in health care it also has cytotoxic, genotoxic, and epigenetic effects on the living systems [15]. Herbal-based silver nanoparticles are indicated to possess wound healing and antibacterial capabilities and are used as an active ingredient in an array of commercially available systemic and topical products. In ancient times, the antidote was directly applied to the site of the ailment; this mode of topical drug administration has been employed in ethnomedicine across different cultures. Topical skin infections are more challenging due to multidrug resistance (MDR) [16]. Consequently, attention has been devoted by formulation scientists to developing new antimicrobial therapeutics with enhanced safety and efficacy. Topical dosage forms are usually made up of polymers or oil-based vehicles intended for skin application, site-specificity, and self-medication [17,18,19].

The objective of the study was to green synthesis and characterize silver nanoparticles, reducing the silver ions of silver nitrate by the extract of Tridax procumbens leaves. Further, these biologically synthesized AgNPs have been investigated for wound healing activity by excision wound model and histopathological studies.

## 2. Materials and Methods

### 2.1. Materials

Silver nitrate (EMSURE^®^) was purchased from Merck, Kenilworth, NJ, USA. Acetic acid, chitosan, triethanolamine, DMSO and, methylparaben were procured from Sigma-Aldrich, St. Louis, MI, USA. All other chemicals and solvents used were of analytical grades and used without further purifications. Ultrapure water used in throughout the experiment was obtained from Fisher Scientific Merck Millipore Milli-Q™ Water Purification System.

### 2.2. Plant Material and Preparation of Tridax Procumbens Leaf-Extract

Fresh leaves of Tridax procumbens, collected from the PSAU university campus, (10 g) thoroughly washed and triturated in mortar and pestle by adding 150 mL of Milli-Q water. The prepared dispersion was then exposed to microwave irradiation for 3 min to accumulate the active constituents. The solution was then filtered using Whatman filter paper (4) in hot conditions to obtain the filtrate of leaf extract to be used in the green synthesis of AgNPs.

### 2.3. Green Synthesis of AgNPs

Freshly prepared aqueous extract (10 mL) was added to 3 mM silver nitrate solution (90 mL) in the Erlenmeyer flask. The mixture was then kept on ceramic-coated aluminum plated themostatistically controlled magnetic stirrer (Magnetic stirrer MS-20D, Witeg Labortechnik, Wertheim, Germany). Dark condition, gentle stirring (50 rpm), 80 °C temperature were maintained and monitored for the synthesis of nanoparticles [20,21]. The reaction was continued until dark brown was achieved, indicating the formation of AgNPs. The colloidal dispersion was then centrifuged at 12,000 rpm for 15 min; sediment was washed many times with deionized double distilled sterile water. The sedimented pellet was lyophilized and preserved in a desiccator for further characterization.

### 2.4. Characterization of Silver Nanoparticles

A total of 6 physicochemical evaluation tests were used to characterize green synthesized AgNPs.

#### 2.4.1. Hydrodynamic Diameter, Polydispersity Index, and Zeta-Potential

Biosynthesized AgNP, characterized by photon correlation spectroscopy using a ZS90 particle analyzer (Malvern Instruments, Malvern, UK). The sample was diluted to 1:200 in Milli-Q water, filled in Zetasizer Cells and Cuvettes (DTS1070). The laser beam crossed with a fixed angle of 90°, and particles under Brownian motion were randomly analyzed for size, heterogeneity, and particle surface charges [22]. Instrument equipped with NanoSight LM10 cell and NanoSight v. 2.3 software for nanoparticle tracking analysis (NTA). Three measurements of each with 20 cycles per sample was performed.

#### 2.4.2. FTIR Spectroscopy

Dried samples under investigation were mounted on the attenuated total reflection crystal. FTIR spectrum was captured 4000 and 400 cm_−1_ using FTIR spectrometer (Thermo Scientific Nicolet iS5, Waltham, MA, USA). Transmittance percentage versus wavenumbers were then plotted and interpreted [23].

#### 2.4.3. XRD Analysis

XRD diffractogram of AgNP was attained by Cu-Kα radiation source in scattering range (2θ) of 20–80 on the instrument operating at a voltage of 45 kV and a current of 40 mA. Intensity (count per second) versus degree 2 theta were plotted, and the crystalline nature of the sample was studied [24].

#### 2.4.4. Scanning Electron Microscopy

Surface morphology examination was conducted by using Scanning electron microscopy (SEM) (SU 8010, Hitachi, Tokyo, Japan), operated at an acceleration voltage of 10 kV. The sample under investigation was coated with a thin layer of gold-palladium by sputter. The image was zoomed and particles were analyzed to determine the morphology of the synthesized AgNPs [25].

#### 2.4.5. BET Analysis

Brunauer–Emmett–Teller analysis was performed by absorption-desorption isotherms using Quantachrome Instruments (Version 5.0, Anton Paar, FL, USA). Approximately 0.2 to 0.4 g of AgNPs were placed in a sample tube and degassed for 2 h using nitrogen to remove moisture and adsorbed gas. The static absorption-desorption isotherms curves plotted by adsorbed gas volume against pressure, in addition to its pore size distribution curve was also plotted by cumulative pore volume vs. average pore radius [26].

#### 2.4.6. TGA and DTG Thermal Analysis

Thermogravimetric (TGA) and Derivative Thermogravimetric (DTG) analysis was performed to understand the loss and decomposition of organic matter to a specific temperature, respectively. TGA study of AgNPs represents thermal stability and composition. [25]. “Netzsch analyzing thermomicro balance” was used to measure the change of mass for AgNPs (sample) as a function of temperature under the controlled environment of heating rate 10 °C/min), gas atmosphere (nitrogen).

### 2.5. Development of AgNPs Loaded Chitosan-Based Topical Gels

Topical gel AgNPs were prepared by dispersing a sufficient amount of chitosan (degree of deacetylation of 76%) into acetic acid 1% solution in order to obtained 2.0% (*w*/*w*) chitosan-based gels. Prepared hydrogels were then mixed with AgNPs (1% *w*/*w*), triethanolamine 0.2% *v*/*w* dimethyl sulfoxide 1% *v*/*w*, and methylparaben 0.02% *w*/*w*. AgNPs loaded chitosan-based gels (AgNPs-CBG) was then mixed with a digitally controlled overhead mixer for overnight to complete the homogenization using a mechanical homogenizer (T-18 Ultra-Turrax, IKA Ltd., Staufen, Germany) [27,28,29]. 

### 2.6. Evaluation of AgNPs Loaded Chitosan-Based Topical Gels

#### 2.6.1. pH Test

The pH value of the topical formulation was assessed by a validated potentiometer fixed electrode. The sample solution was prepared by dispersing 1 gm of AgNPs in deionized-purified water of 10 mL. The electrode was then dipped, and reading was noted, and the measurement of the sample was performed in triplicate [30].

#### 2.6.2. Temperature Cycling and Centrifugal Test

T, tested for phase separation. In this test, the AgNPs loaded chitosan chitosan-based gels (1 gm) subjected to freezing and thawing 2 days’ cycles at 45 °C (Hot) and 4 °C (Cold). Besides, centrifugal testing was performed by taking the sample (1 gm) in Eppendorf tube, kept it in the rotor sample holder, centrifuged at 3000 rpm for 30 min at 6 °C, and observed for sediment in the bottom [28].

#### 2.6.3. Spreadability Test

A spreadability test was performed by taking the sample (AgNPs loaded chitosan-based gels—AgNPs-CBG) under study sandwiched between two glass plates of (10 cm × 20 cm) size. Thereafter, 50 g weight was placed over the top plate, kept for 60 s. The spreading diameter of AgNPs-CBG was measured thrice, and standard deviation was calculated [31].

### 2.7. Antibacterial Study

Antibacterial effects of developed AgNPs loaded chitosan-based gel were assessed against *Bacillus subtilis* (ATCC 11774) Gram-positive and *Klebsiella pneumoniae* (NCTC 9633) Gram-negative bacterial strains. Disk diffusion technique was used in which each disk was filled with AgNP CBG, different concentrations of the sample (20–100%), and distilled water (Blank). Then, the plates were incubated for 2 days at 37 °C, and the zone of inhibition (mm) was measured for both bacterial strains [27].

### 2.8. Biocompatibility Study

The biocompatibility test was performed according to Muqtader et al. The hemolytic activity of all treatments was measured using a hemoglobin release assay by spectrophotometer. In vitro hemobiocompatibility assessment of precursors (AgNPs and chitosan) and AgNPs loaded chitosan-based gels (AgNP-CBG) was performed by measuring the absorbance at 540 nm for the hemoglobin released in aliquots. Percentage of hemolysis was performed by taking sod dodecyl sulfate, and DMSO act as positive and negative controls, respectively. Summarily, EDTA-treated RBCs were washed with PBS and centrifuged for 20 min at 1500× *g*. Sedimented erythrocytes (400 µL) was resuspended in PBS (1 mL) and treated with each sample separately. Subsequently, the cells were treated and incubated for one hour at 37 °C. The treated samples were then centrifuged at 795× *g* for 20 min, the absorbance of supernatant (AgNPs, Chitosan, AgNPs-CBG) was measured, and hemolysis (%) was calculated using the following equation [29].

$$Hemolysis\;\left(\%\right)=\frac{{\mathrm{Absorbance}\;}_{sample}-{\mathrm{Absorbance}\;}_{negative}}{{\mathrm{Absorbance}\;}_{positive}-{\mathrm{Absorbance}\;}_{negative}}\times 100$$

### 2.9. In-Vivo Excision Model Wound Healing Study

Wound healing activity was performed in animals obtained from the animal care unit. The approval to carry out the study was granted by Bioethical Research Committee (BERC-000–04-21; date: 06-04-2021), Prince Sattam bin Abdulaziz University, Al-Kharj, Saudi Arabia.

Animals: three groups comprising negative control, standard, and test (AgNPs) with 8 mice in each group anesthetized using diethyl ether. The back of the mice was shaved. Under an aseptic environment, an approximately 4.5 cm^2^ cutaneous wound with full-thickness deepened down to adipose tissue was made at the dorsum using a sterile stainless steel scalpel blade. The wound was left untreated besides applying standard (Silver sulfadiazine 1% cream) and AgNPs—loaded CBG once a day until complete healing was observed. The wound areas were measured every week until 21 days, and wound contraction was calculated as a percentage of reduction in the wound area as given in equation-1. Epithelialization time was recorded as the number of days needed after wound infliction for the scab to fall off, leaving no raw wounds behind [27].

$$\mathrm{Wound contraction}\left(\%\right)=\;\frac{\left[\mathrm{Initial day wound area}-\mathrm{Specific day wound area}\right]}{\mathrm{Initial day wound area}}\;\times \;100$$

### 2.10. Histopathological Study

Mice skin wound tissue samples (3–5 cm) from 3 different groups (negative control, standard and test) collected and immersed immediately in formalin (10%) and then processed “(ASP300 s, Leica Biosystems, Buffalo Grove, IL, USA)”. Thereafter, processed tissue samples were fixed in paraffin wax and 5 µm thickness section was prepared using rotary microtome “(SHUR/Cut 4500, TBS, Durham, NC, USA).” Three sections of each sample were stained using hematoxylin and eosin technique, Masson trichrome, and Verhoeff special staining methods for visualizing cell structure and connective tissue fibers [32,33].

In the hematoxylin and eosin method, the deparaffinize sections were rehydrated through descending grades of ethanol to water, removing fixation pigments if necessary. The section was immersed in hematoxylin “(HX082464, MERK, Darmstadl, Germany)” for 10 min. Wash well in running tap water until sections ‘blue’ for 5–10 min or less. Stain in 1% eosin Y for 10 min. Wash in running tap water for 1–5 min. Dehydrate through ascending ethanol, clear, and mount in a mixture of distyrene, a plasticizer, and xylene (DPX). Masson trichrome technique for connective tissue fibers demonstration (mainly collagen) was performed using Stain nuclei with “Weigert’s iron hematoxylin” for 10 min, wash with water, stain in an acid fuchsin solution for 5 min, rinse rapidly in water, differentiate in 1% phosphomolybdic acid for approximately 5 min, drain and counterstain with methyl blue, dehydrate, clear and mount sections in DPX. Verhoeff technique for connective tissue fibers demonstration (mainly elastic fibers) was performed using stain reticular fibers with “Verhoeff’s hematoxylin” for 30 min, wash in tap water, differentiate in 2% ferric chloride solution, check microscopically for black fibers on a gray background, rinse in water, counterstain eosin for 5 min, dehydrate, clear and mount sections in DPX [33].

## 3. Results

### 3.1. Characterization of Silver Nanoparticles

#### 3.1.1. Hydrodynamic Diameter, Polydispersity Index, and Zeta-Potential

**Figure 1:**
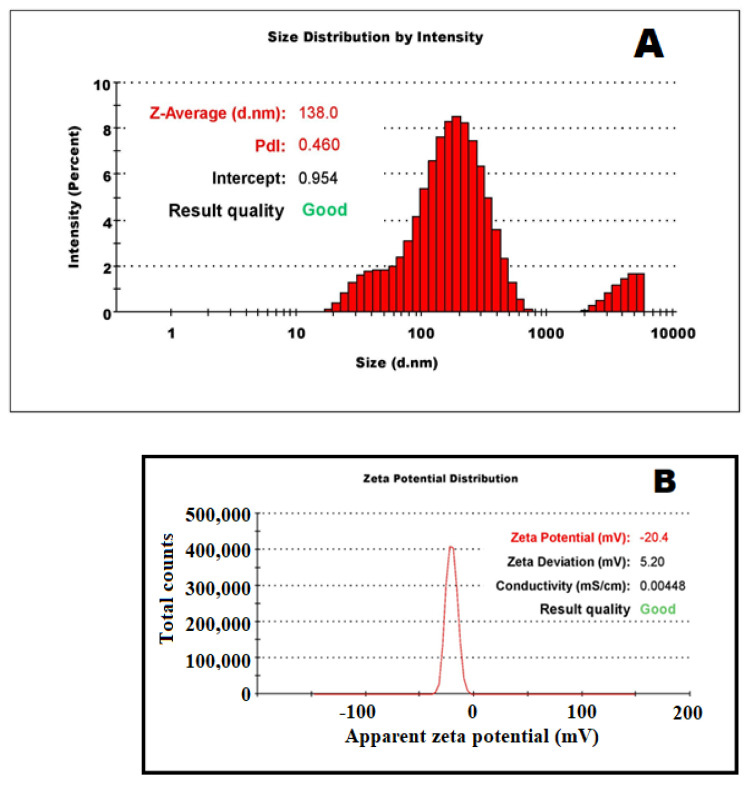
Photon correlation spectroscopy (PCS) of the silver nanoparticles. (**A**) Hydrodynamic particle size distribution and PDI, (**B**) zeta potential.

Photon correlation spectroscopy is a non-destructive technique that measures the light scattered from a laser that passes through a sample to measure the hydrodynamic size of particles, PDI, and zeta-potential, as shown in Figure 1. Particle size was found to be 138.0 ± 2.1 nm with a relatively monodispersed distribution (polydispersity index: 0.460 ± 0.3). The surface of the particles was profound to be negatively charged with a zeta potential of −20.4 ± 5.20 mV, indicating optimum inter-particle repulsion and does not allow the agglomeration-related instability. The size obtained in photon correlation spectroscopy is usually more significant than the SEM because of sample suspended in liquid as colloidal dispersion and under Brownian motion [34].

#### 3.1.2. FTIR Spectroscopy

**Figure 2:**
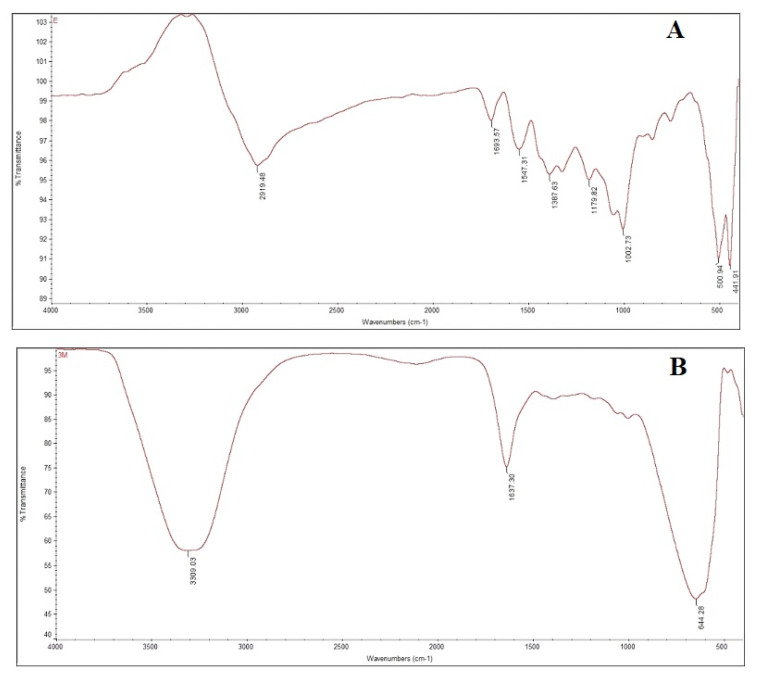
FTIR spectrums of Tridax procumbens leaf powder extract (**A**) and green synthesized silver nanoparticles (**B** = AgNPs obtained by reacting leaf extract and 1 × 10^−3^ M AgNO_3_ solution).

FTIR spectrum (Figure 2A) of the extract showed the peaks at 2919.48, 1693.57, 1547.31, 1387.63, 1179.82 cm^−1^, corresponding to the aliphatic (C-H) stretching vibration of hydrocarbon chains, amides (N-H) stretching in addition to peptide bond, stretching vibration of ethylene (C=C) and stretching in a carboxylic acid (C=O) such as functional groups, respectively. The absorption peak at 1002.73 cm^−1^ is due to the presence of aliphatic amine (C-N) stretching vibrations. The spectrum of AgNPs (Figure 2A,B) indicated prominent peaks at 3309.03 and 1637.30 cm^−1^ functioning of reduction and stabilization of silver nanoparticles by Tridax procumbens [35].

#### 3.1.3. XRD Analysis

**Figure 3:**
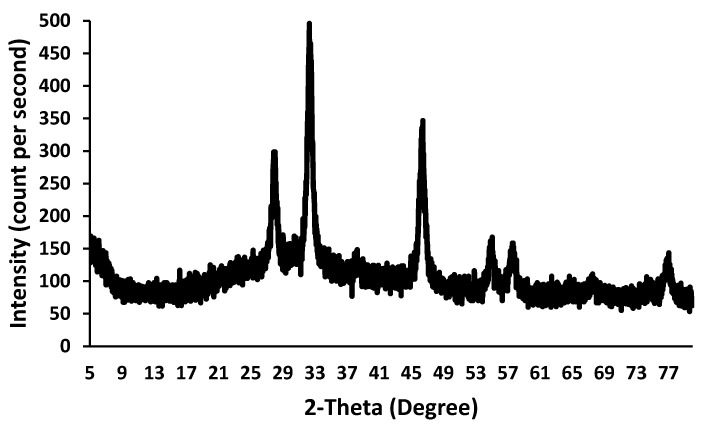
X-ray diffraction patterns of silver nanoparticles green synthesized in Tridax procumbens L aqueous extract using 1 × 10^−3^ M AgNO_3_ solution.

XRD diffractograms of biosynthesized AgNPs showed the crystalline nature of the nanoparticle. The high peaks in the XRD spectrum confirmed that the prepared AgNPs were in nanocrystal and crystalline form. The XRD peaks, as shown in Figure 3, explain the number of Bragg reflections to the planes (122), (111), (200), (220), and (311) facet of silver crystal at 2θ values of 37°, 47°, 65°, and 77°, respectively. Other researchers also reported these results and data. The peaks reflected other than these were at 33° and 57°, which could be due to the crystalline organic constituent [36].

#### 3.1.4. Scanning Electron Microscopy

**Figure 4:**
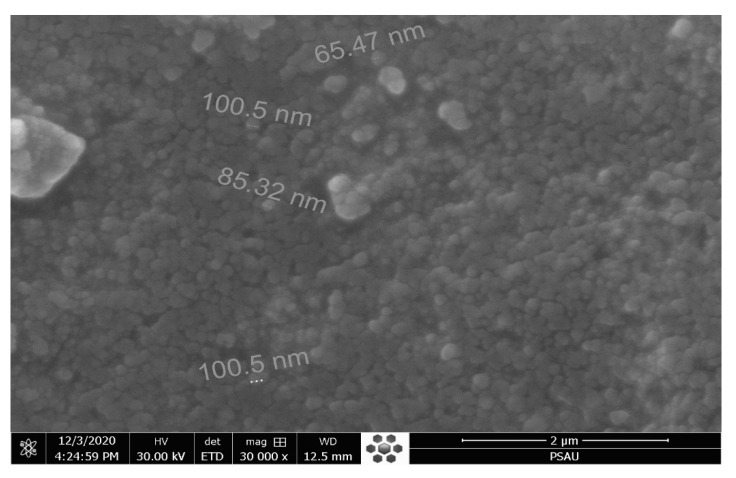
SEM images of silver nanoparticles green synthesized using leaf extracts of Tridax procumbens.

SEM analysis revealed the size and shape of green-synthesized AgNPs. The micrographs of AgNPs reflect that prepared NPs were spherical with some aggregates and represented a high-density crystalline nature. Spherical and slight truncated morphology could be due to the capping of NPs by the chemical constituents present in the extract of T.P leaves. The scale on the particles measured the size range between 65–100 nm, indicating polydispersity. The micro-picture of sample AgNPs is shown in Figure 4. Consistent with the previous reports, SEM examination of AgNPs showed spherical and semi-spherical shapes [37].

#### 3.1.5. BET Analysis

**Figure 5:**
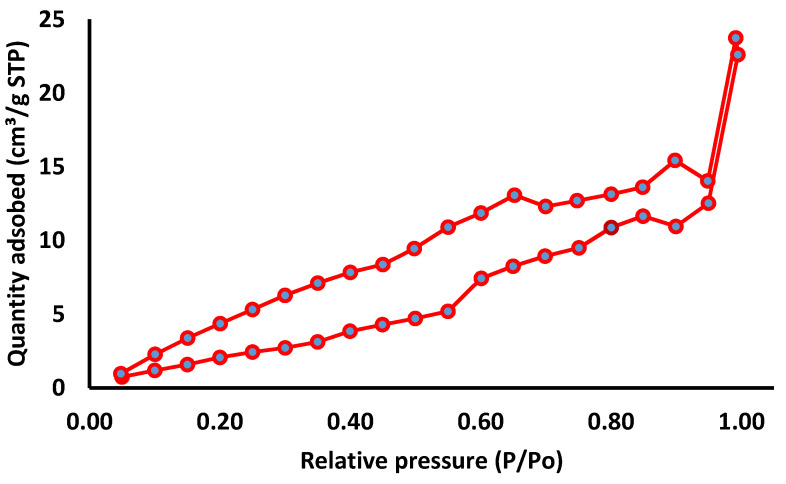
Nitrogen absorption-desorption isotherms of the green synthesized Tridax procumbens capped AgNPs at (77.5 K).

**Figure 6:**
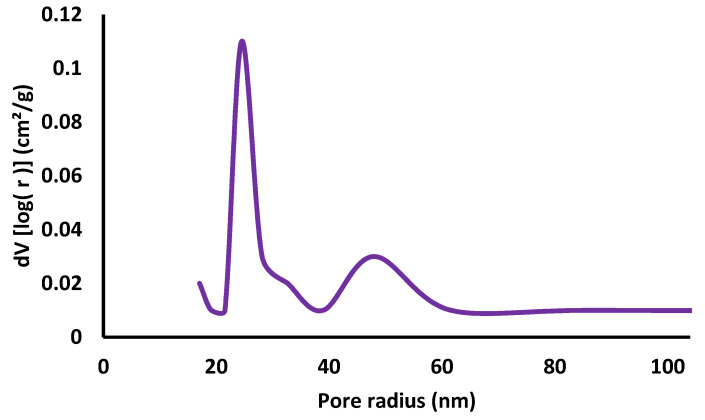
BJH pore size distribution curves of AgNPs green synthesized using Tridax procumbens aqueous leaf extract.

The absorption-desorption isotherms of the samples AgNPs shown in Figure 5 presents the type IV hysteresis phenomenon interpreted to be Slit-like pores in AgNPs pair combined with a type II isotherm presumed to be without pores. Based on the Barrett–Joiner–Halenda (BJH) model, the results were found to be surface area (12.861 m^2^/g), pore volume (0.037 cc/g), and pore radius (24.50 nm) [38]. BJH pore size distribution curves of AgNPs are shown in Figure 6. From the curve, it was evident that most of the particles have a pore size of less than 40 nm; the smallest pore size could be 24 nm; the results indicate narrow pore size distribution. Moreover, such micropores have not been seen in SEM micrographs, Figure 4.

#### 3.1.6. TGA and DTG Thermal Analysis

**Figure 7:**
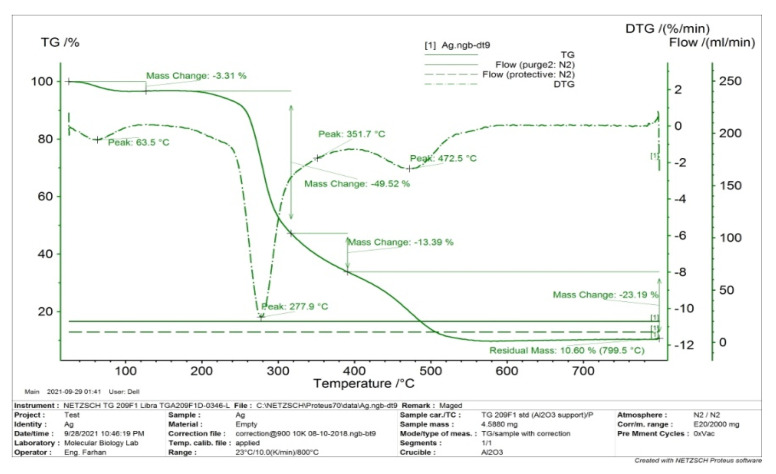
TGA and DTG thermal spectrum of green synthesized AgNP.

Thermal properties TGA and DTG spectra are shown in Figure 7. The sample (AgNPs) was studied at a temperature range from 23 °C to 799.5 °C using a simultaneous thermal system. It was observed from the TGA curve that dominant weight loss of the sample occurred in the temperature region between 200 and 300 °C due to the evaporation of moisture and volatile organic content. There is almost no weight loss below 200 °C and above 500 °C. There was nearly no degradation above 500 °C that accounts for the weight of silver; the residual mass was 10.60% at 799.5 °C. The degradation pattern of organic components was observed in the temperature ranged between 100–500 °C. Overall, TGA results show a loss of 13.39% up to 300 °C. DTG plot displays a sharp endothermic peak between 200 °C and 300 °C, which could be attributed to the crystallization of AgNPs. DTG profiles indicate complete simultaneous thermal decomposition and crystallization of the sample.

### 3.2. Development of AgNPs Loaded Chitosan-Based Topical Gels

Prepared AgNPs loaded chitosan-based gels were observed to be clear and free from gritty particles.

### 3.3. Evaluation of AgNPs Loaded Chitosan-Based Topical Gels

pH test of the gel showed the value (pH 6.2), if the sample pH ranged from 6–7.4, the formulation was considered to be compatible. Based on the result, it is evident that the prepared topical gel formulation was skin compatible and may not cause any dermal irritation.

The topical formulations submitted to thermal and centrifugal tests showed good physical stability, with no phase separation and sedimentation under the controlled experimental conditions.

The spreading ability of topical gels indicates the smearing of the formulation after application over the applied area. Increased spreadability was the desired quality for ease of application and efficiency of preparation. AgNPs CBG showed 18 mm spreading diameter, exhibited the extent to which the topical gel readily spread on the application area by low shear [39].

### 3.4. Antibacterial Study

**Figure 8:**
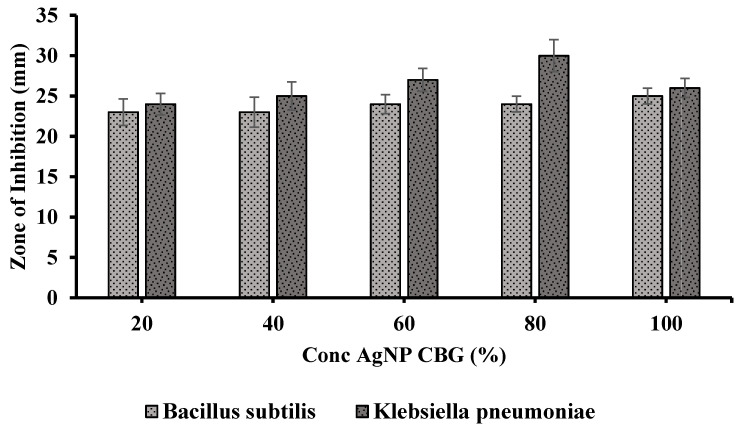
Antimicrobial activity of Ag NPs loaded chitosan gel against *Bacillus subtilis* gram (+) and *Klebsiella pneumoniae* gram (−).

**Figure 9:**
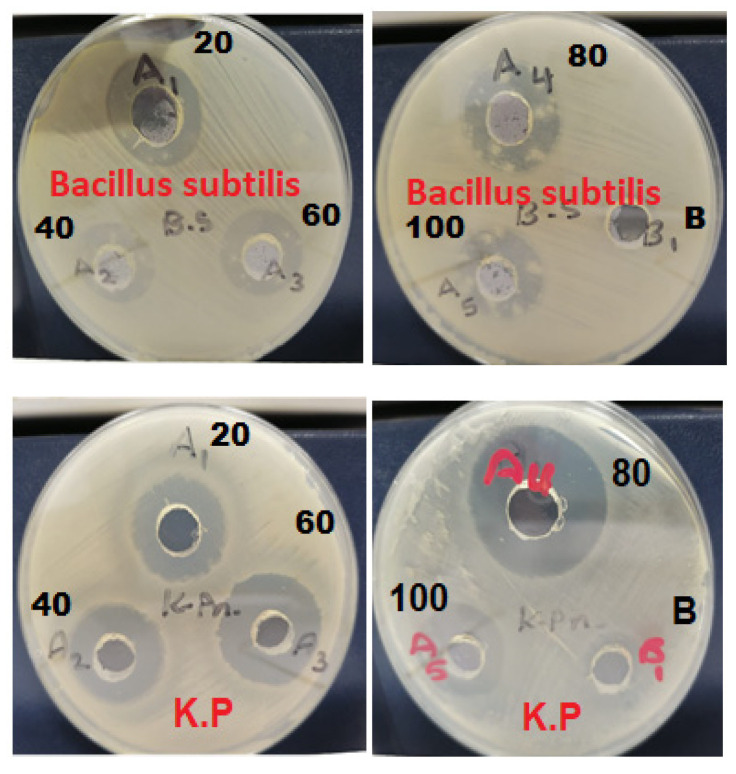
Concentration-dependent (20–100%) zone of inhibition (mm) of AgNPs loaded chitosan gel against *Bacillus subtilis* gram (+) and *Klebsiella pneumoniae* gram (−).

AgNPs prepared by green synthesis from T. procumbens showed antibacterial activity against both Gram-negative and +positive bacterial strains. Dose-dependent efficacy was observed, the results were supported by previous studies reflecting more potency of the AgNPs against Gram-negative compared to the Gram-positive bacteria. The zone of inhibition was found to be (23 ± 1 to 25 ± 0.98 mm) and (24 ± 1 to 30 ± 1) at 20–100% concentration for *Bacillus subtilis* and *Klebsiella pneumoniae*, respectively (Figure 8 and Figure 9). The green synthesized AgNPs loaded chitosan gels has therapeutic efficacy, and proved to be a potent topical antimicrobial formulation against Gram-positive and Gram-negative organisms. The wound will harbor microbial strains henceforth, and the developed gel application could enhance wound healing by eradicating the infection.

### 3.5. Biocompatibility Study

**Figure 10:**
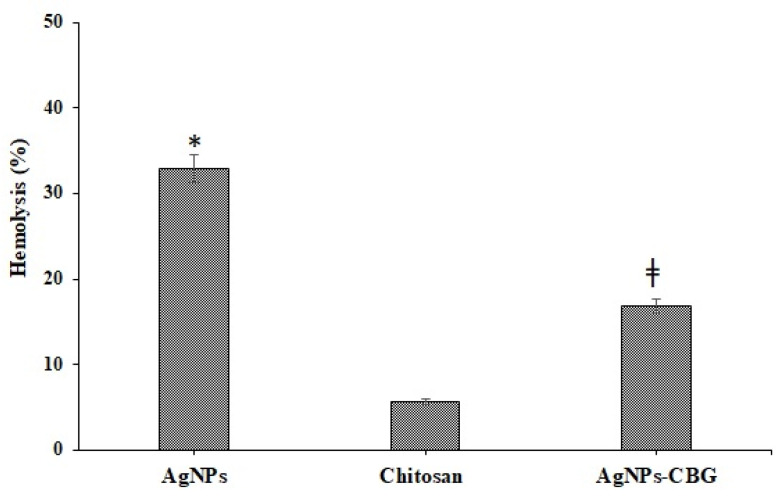
Hemolysis (%) AgNPs, Chitosan, and AgNPs loaded chitosan gels. * *p* < 0.005 highly significant when compared between AgNPs vs pure chitosan and ǂ < 0.05 significant when compared between AgNPs-CBG vs pure chitosan.

Comparable AgNPs loaded chitosan-based gels (1% *w*/*w*) had better protective effects on the RBCs by preventing cell destruction, a process called hemolysis. Hemolysis assessment was considered a simple, rapid, and reproducible test to study the biocompatibility of the developed formulations with the skin layers and blood. Based on Figure 9, we found that AgNPs alone have high hemolytic activity compared to chitosan and AgNP CBG. The percentage of hemolysis was found to be (32.87 ± 2.34%, 5.67 ± 1.98%, 16.88 ± 2.89%) for AgNPs, chitosan, and AgNP CBG, respectively (Figure 10). Therefore, the developed AgNPs loaded chitosan gels could be considered non-toxic and biocompatible for topical dermal application.

### 3.6. In-Vivo Excision Model Wound Healing Study

**Figure 11:**
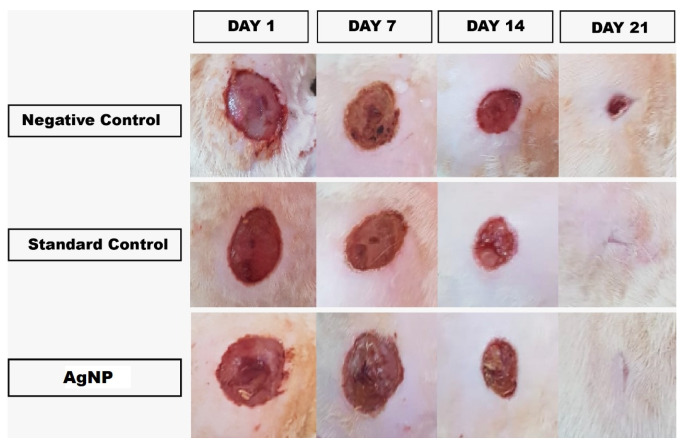
Progress of cutaneous wound healing in negative-control mice untreated, standard control mice treated with silver sulfadiazine (1% cream), and experimental mice treated with Tridax procumbens capped AgNPs loaded chitosan-based gels at days 1, 7, 14, and 21 post wounding.

**Figure 12:**
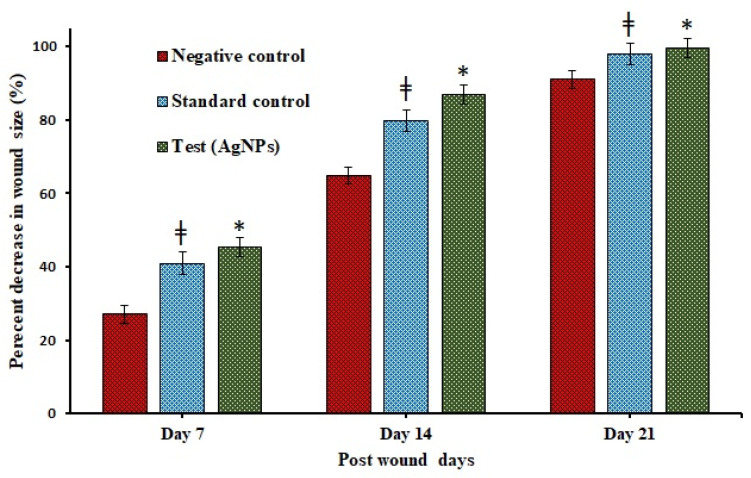
Percentage decrease in wound size over in negative control, standard control, and test group treated with AgNPs loaded chitosan-based gels over a period 7, 14 and, 21 days, measured morphometrically. * *p* < 0.005 highly significant when compared between test (AgNPs) vs. negative control and ǂ < 0.05 significant when compared between test (AgNPs) vs. standard control.

The results of wound healing activity and epithelialization reflected in Figure 11; Figure 12 showed a significant progressive healing process in the test-group of animals treated with AgNPs compared with those who received negative control (placebo) and standard (Silver sulfadiazine 1%). After the injury, the oozing of blood will occur, followed by clot formation and synthesis of ground substances, mainly composed of proteoglycans—a non-fibrillary component. Proteoglycans, along with glycosaminoglycan—a protein core linked covalently to linear heteropolysaccharides actively participate in the wound healing process [40].

Moreover, epithelialization data represents; (27.09 ± 1.12%, 67.79 ± 2.54%, 91.03 ± 1.32%), (40.92 ± 1.76%,79.78 ± 1.99%,98.01 ± 1.68%), (45.49 ± 1.23%, 86.86 ± 1.87%, 99.69 ± 1.32%) percentage decrease of contraction in the wound size for negative control (placebo), standard (Silver sulfadiazine 1%) and test (AgNPs-CBG) at 7, 14, and 21 days, respectively.

### 3.7. Histopathological Study

**Figure 13:**
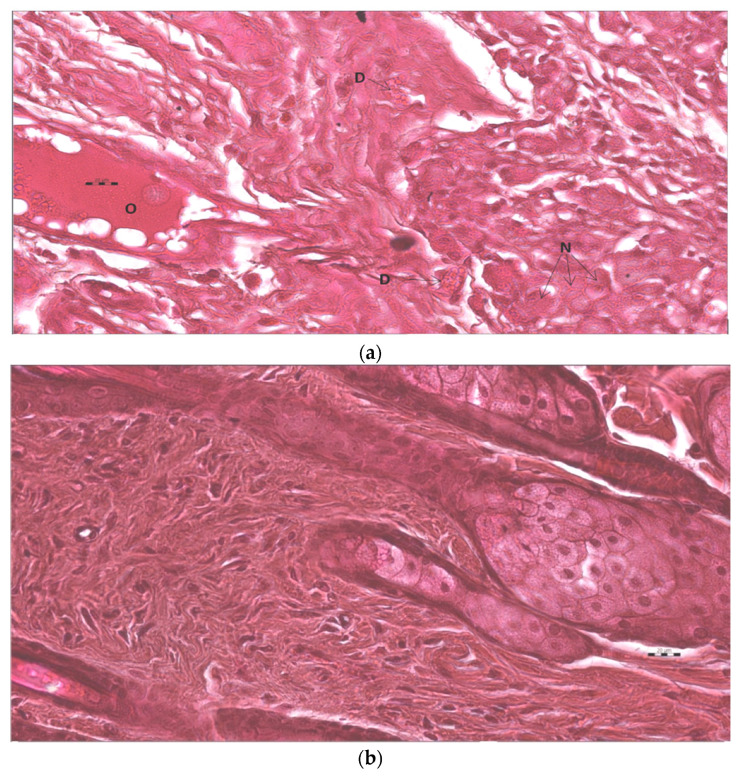

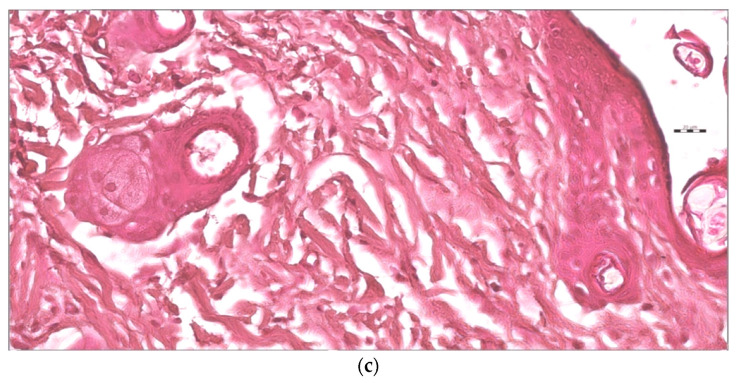
(**a**) This is micro photo of negative control show severe tissue damage in the form of necrosis due to the absence of nuclei (N), degeneration in form of vacuoles (D), as well as occlusion of blood vessels by large spots of hyaline material (O). Stain is H&E, magnification is 400×, and scale bar is 20 µm. (**b**). The standard sample shows an almost normal tissue sample. Stain is H&E, magnification is 400×, and the scale bar is 20 µm. (**c**). Test tissue samples treated with AgNPs loaded chitosan-based gels show almost normal tissue sample. Stain is H&E, magnification is 400×, and scale bar is 20 µm.

**Figure 14:**
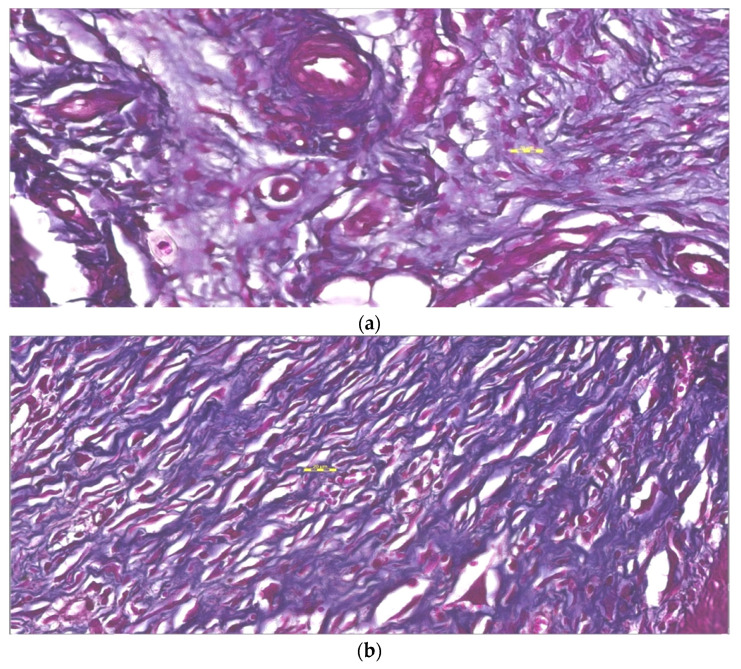

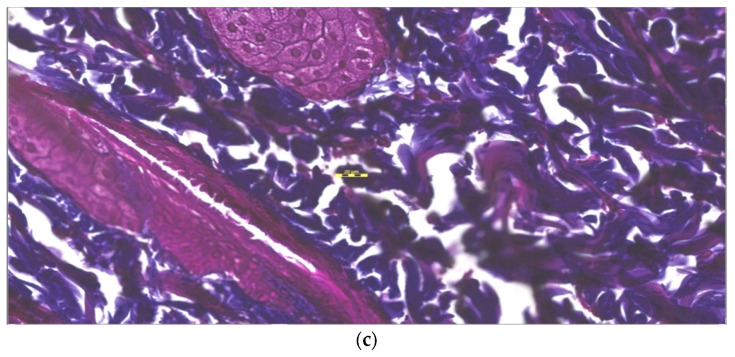
(**a**) Negative control sample shows loss of collagen fibers as light blue color. Stain is Masson trichrome, magnification is 400×, and the scale bar is 20 µm. (**b**). The standard sample shows improved status of collagen fibers as dark blue color. Stain is Masson trichrome, magnification is 400× and scale bar is 20 µm. (**c**). Test tissue samples treated with AgNPs loaded chitosan-based gels show almost normal and much improve status of collagen fibers. Stain is Masson trichrome, magnification is 400× and scale bar is 20 µm.

**Figure 15:**
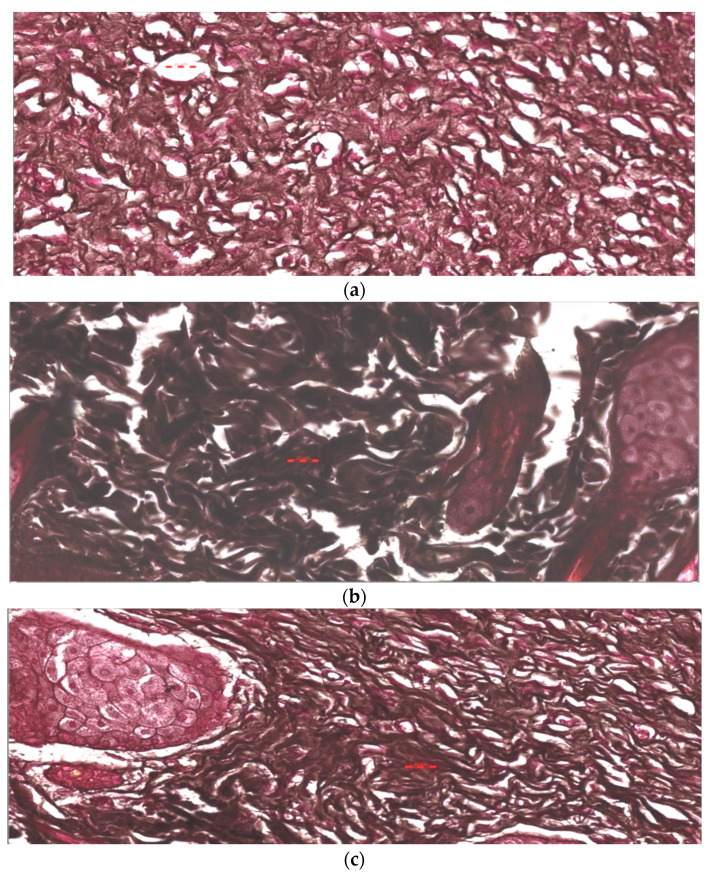
(**a**). Negative control sample shows loss of elastic fibers as less density of black color. Stain is Verhoeff method for elastic fibers, magnification is 400×, and the scale bar is 20 µm. (**b**). Standard samples show an improved status of elastic fibers as increased density of black color. Stain is Verhoeff method for elastic fibers, magnification is 400×, and the scale bar is 20 µm. (**c**). Test tissue samples treated with AgNPs loaded chitosan-based gels show an improved status of elastic fibers as increased density of black color. Stain is Verhoeff method for elastic fibers, magnification is 400×, and the scale bar is 20 µm.

Histopathological study of wounded animal tissue treated (test) and untreated (control) shown in Figure 13a–c for hematoxylin and eosin examination. Tissue from the control (untreated) stained with H&E represents severe damage and dead cells. It also showed the absence of nuclei and occlusive blood vessels indicated by a blood clot and hyaline substances in blood vessels with the hemorrhagic opening of blood vessels. The results from the standard (Silver sulfadiazine 1% cream) showed improvement but still have necrosis and scattered red blood cells, indicating hemorrhage. However, the test group (AgNPs—Chitosan-based gels) showed complete healing of wounds with almost normal dermal structure. Tissue untreated (control) stained with MT stain showed yellow photomicrograph (Figure 14a–c) indicating loss of collagen fibers (light blue color), whereas the tissue applied by topical standard and test formulation show improved collagen fiber (dark blue color) and normal skin structure. The third stained process used in the study was the Verhoeff method (Figure 15a–c), in which control (negative) tissue showed less elastic fibers and treated tissue with the standard (Silver sulfadiazine 1%) and test (AgNPs-CBS) indicated improved density of elastic fibers. The order of increased elastic fiber is tested > standard > negative control group. A hypothetical mechanism involved in the AgNP wound healing activity could be silver NPs initiative of fibroblasts differentiation into myofibroblasts, which in turn accelerates the wound contraction, proliferation, and relocation of keratinocytes.

The epidermal layer starts healing and recovering in the AgNPs treated group compared to the untreated group. Hemorrhagic area with infiltrated inflammatory cells numbers was reduced in AgNPs loaded CBG applied treated group. After the 3-week treatment with AgNPs–CBG, tissue restructures and restored stratified epidermis with granular and cornified layers indicating the efficacy of AgNPs in wound healing. Histopathological study using H&E method, Masson trichrome method, and Verhoeff method indicated the most improvement were in the AgNPs–CBG treated test group [33]. Our results were in agreement with reflecting that AgNPs could accelerate the granulation of tissue [41,42,43]. Developed gels completely healed the wound with the early progress of the primary scar of collagen and rudimentary cutaneous appendages.

## 4. Conclusions

The development of topical AgNPs loaded chitosan-based gels require precise and multiple characterizations to ensure effective and safe alternative dosage form for smart, ecofriendly, highly effective, and multi-dimension wound healing nano-systems. The current study demonstrates the green synthesis of AgNPs from Tridax procumbens leaves and the physicochemical characterization of AgNPs. The results confirmed that AgNPs were successfully green synthesized with nanosize range, monodisperse, antiadhérent crystalline properties, and spherical shape. Chitosan-based gel loaded with AgNPs showed broad-spectrum antibacterial activity along with biocompatible efficiency. Moreover, the topical gels indicated potential application in the management of wounds with high microbial bioburden.

## Data Availability

The data presented in this study are available on request from the corresponding author.
